# A Novel Homozygous p.R1105X Mutation of the *AP4E1* Gene in Twins with Hereditary Spastic Paraplegia and Mycobacterial Disease

**DOI:** 10.1371/journal.pone.0058286

**Published:** 2013-03-05

**Authors:** Xiao-Fei Kong, Aziz Bousfiha, Abdelfettah Rouissi, Yuval Itan, Avinash Abhyankar, Vanessa Bryant, Satoshi Okada, Fatima Ailal, Jacinta Bustamante, Jean-Laurent Casanova, Jennifer Hirst, Stéphanie Boisson-Dupuis

**Affiliations:** 1 St. Giles Laboratory of Human Genetics of Infectious Diseases, Rockefeller Branch, The Rockefeller University, New York, New York, United States of America; 2 Clinical Immunology Unit, Department of Pediatrics, King Hassan II University, Ibn-Rochd Hospital, Casablanca, Morocco; 3 Pédiatre du Secteur Libéral, neurologie pédiatre, Casablanca, Morocco; 4 Laboratory of Human Genetics of Infectious Diseases, Necker Branch, U980, Institut National de la Santé et de la Recherche Médicale (INSERM), Paris, France; 5 University Paris Descartes, Paris Cité Sorbonne, Necker Medical School, Paris, France; 6 Study Center for Primary Immunodeficiencies, Assistance Publique-Hôpitaux de Paris (AP-HP), Necker Hospital, Paris, France; 7 Pediatric Immunology-Hematology Unit, Necker Hospital, Paris, France; 8 Cambridge Institute for Medical Research, University of Cambridge, England, UK; University Hospital Vall d’Hebron, Spain

## Abstract

We report identical twins with intellectual disability, progressive spastic paraplegia and short stature, born to a consanguineous family. Intriguingly, both children presented with lymphadenitis caused by the live Bacillus Calmette-Guérin (BCG) vaccine. Two syndromes – hereditary spastic paraplegia (HSP) and mycobacterial disease – thus occurred simultaneously. Whole-exome sequencing (WES) revealed a homozygous nonsense mutation (p.R1105X) of the *AP4E1* gene, which was confirmed by Sanger sequencing. The p.R1105X mutation has no effect on *AP4E1* mRNA levels, but results in lower levels of AP-4ε protein and of the other components of the AP-4 complex, as shown by western blotting, immunoprecipitation and immunofluorescence. Thus, the C-terminal part of the AP-4ε subunit plays an important role in maintaining the integrity of the AP-4 complex. No abnormalities of the IL-12/IFN-γ axis or oxidative burst pathways were identified. In conclusion, we identified twins with autosomal recessive AP-4 deficiency associated with HSP and mycobacterial disease, suggesting that AP-4 may play important role in the neurological and immunological systems.

## Introduction

Hereditary spastic paraplegia (HSP) constitutes a large, genetically diverse group of inherited neurologic disorders characterized by progressive spasticity and weakness of the lower limbs [1]. HSP is uncommon, but not rare, with a prevalence of ∼3–9/100,000 in most populations [1], [2]. Inheritance may be X-linked recessive, autosomal recessive or dominant, and age at onset varies widely, from early childhood to adulthood [1], [3]. HSP has historically been classified as ‘pure’ or ‘complicated’ on the basis of the absence (pure) or presence (complicated) of associated clinical features, such as distal amyotrophy, cognitive dysfunction, retinopathy, cataracts, ataxia, thin corpus callosum, peripheral neuropathy and deafness [1], [2]. However, HSP is increasingly being classified genetically, as genetic mapping has identified at least 52 different HSP loci, designated SPG (for spastic paraplegia) 1 through 52, in order of discovery [3]. In particular, mutations of the four genes encoding the subunits of the AP-4 (adaptor protein-4) complex have recently been identified in patients with autosomal recessive spastic paraplegia [4], [5], [6], [7], [8], [9]. The four subunits of the AP-4 complex have the following OMIM (http://omim.org/) classifications: *AP4B1*, SPG47 (OMIM*614066); *AP4M1*, SPG50 (OMIM*612936); *AP4E1* SPG51 (OMIM*613744) and *AP4S1*, SPG52 (OMIM*614067) [3], [10]. AP-4 is one of a family of five closely related protein complexes that are ubiquitously expressed, and function in vesicle trafficking, in which they select cargos for inclusion in transport intermediates [9], [10], [11]. AP-4 consists of two large subunits (β4 and ε), one medium (μ4) and one small (σ4) subunit. The N-terminal domains of the large adaptins, together with the μ and σ subunits, form the AP ‘core’, and the C-terminal ‘appendage’ domains of the large subunits form binding platforms for the recruitment of additional coat proteins. The five AP complexes have different subcellular distributions and mediate different transport steps [9].

Susceptibility to infection with weakly virulent mycobacteria, including BCG and nontuberculous mycobacteria (or environmental mycobacteria), may be seen as one of the symptoms in patients with primary or acquired immune deficiencies, including chronic granulomatous disease [12] and classical severe combined immune deficiency [13], immunocompromised patients with HIV infection [14] or cystic fibrosis patients [15]. However, the infectious phenotype of these patients is not restricted to mycobacteria. By contrast, the syndrome of Mendelian susceptibility to mycobacterial disease (MSMD#OMIM209950) is a rare congenital disorder characterized by clinical disease caused by weakly virulent mycobacteria, such as BCG vaccines or environmental mycobacteria, in otherwise healthy children [16], [17]. Since the identification of the first genetic etiology of this syndrome in 1996, nine morbid genes (*IFNGR1*, *IFNGR2*, *IRF8*, *STAT1*, *CYBB*, *NEMO*, *IL12B*, *IL12RB1* and *ISG15*) and 17 different genetic etiologies of MSMD have been discovered. All these defects impair the production of, or the response to IFN-γ either directly or indirectly [17], [18], [19], [20], [21], [22], [23], [24], [25]. The search for new morbid genes by whole-exome sequencing (WES) has already proved successful in other infectious diseases, even when applied to a single family [26], [27]. We report here twins born to a consanguineous family presenting with early-onset, complex spastic paraplegia and mycobacterial disease. We used WES to search for genetic lesions in these twins with both neurological and immunological disorders.

## Patients and Methods

### Case Reports

**Figure 1:**
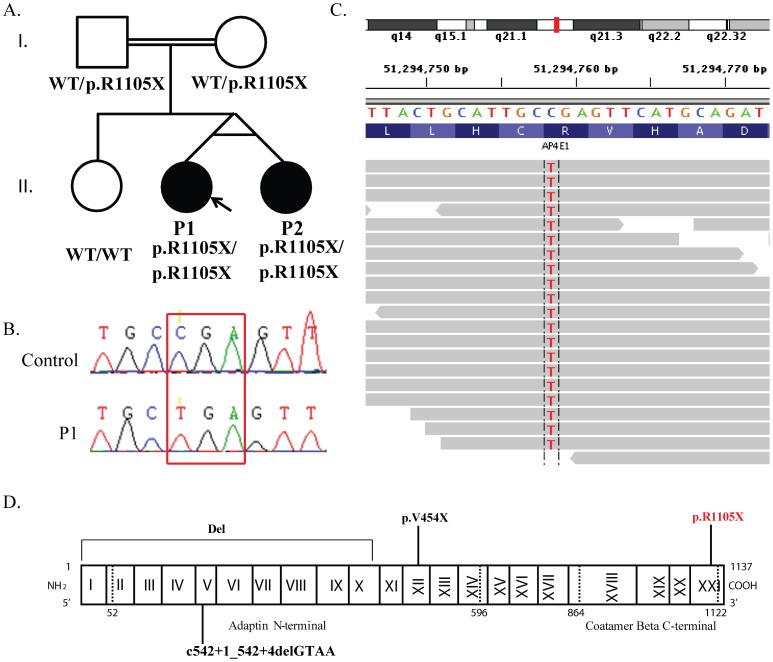
Pedigree and *AP4E1* mutation in P1 and P2. A). Pedigree of the family. The segregation of the *AP4E1* p.R1105X mutation is also indicated. B). Electrophoregram showing the homozygous mutation of P1 with respect to the control sequence. C). Illumina sequencing reads displayed for patient P1. D). Schematic diagram of the structure of AP-4ε, with the delimitation and numbering of the corresponding exons. The other known mutations of the *AP4E1* gene and the mutation described here (in red) are also indicated.

The two patients (P1 and P2) are identical twins born to first-cousin Moroccan parents (Figure 1A). No complications were observed during the pregnancy, but neonatal asphyxiation was noted during the delivery. When examined at the age of three years, the twins displayed marked developmental retardation, including microcephaly, an inability to walk unaided, abnormal speech and abnormal circadian development. They kept smiling or laughing for no obvious reason. Abnormal drooling was also observed. Both patients had muscular hypotonia. No seizure or involuntary movement was observed in either of the patients. Both patients presented discreet but remarkable facial gestalt with a prominent, bulbous nose, a wide mouth and coarse features. Neurological examination revealed spastic paraplegia of the lower extremities. Denver II assessments of both children demonstrated a significant delay in the acquisition of major skills, such as motor and adaptive skills, language and social skills, at the age of three. Both patients were of short stature and had a low body weight. Their clinical features are summarized in detail in Table 1. Electroencephalography (EEG) revealed a diffusive slow wave (theta and delta wave). Based on these findings, both twins were diagnosed with type I complex HSP.

**Table 1:** Summary of clinical neurological presentations.

Patient	P1	P2
**Subunit**	AP4E1	AP4E1
**Sex**	F	F
**Age at onset**	Infancy	Infancy
**Age at observation (years)**	3	3
**Head circumference**	46 cm (−2SD)	45.5 cm (−2.5SD)
**Mental retardation**	+++	+++
**Contractures**	–	–
**Early infantile hypotonia**	+	+
**Spastic tetraplegia**	Lower extremities only	Lower extremities only
**Hypertonia**	–	–
**Hyperreflexia**	–	–
**Babinski sign**	–	–
**Extensor plantar reflex**	–	–
**Epilepsy**	–	–
**Deambulation**	Never achieved	Never achieved
**Normal speech**	Never acquired	Never acquired
**Sphincter control**	Absent	Absent
**Eye evaluation**	Slight myopia and astigmatism	Slight astigmatism
**Hearing evaluation**	Transmission deafness on left side, normal on right side	–
**Neuroimaging (CT)**	Atrophy of the inferior vermis with cortical atrophy	Atrophy of the inferior vermis with cortical atrophy
**Loss of acquired function**	++	++
**Purposeful hand use**	++	++
**Pseudobulbar signs**	Drooling+++, stereotypic laughter+++, jaw jerk++, gag reflex++	Drooling+++, stereotypic laughter+++, jaw jerk++, gag reflex++

In addition to neurologic problems, both patients presented unilaterally enlarged and inflammatory axillary lymph nodes at nine months of age. Both had been vaccinated with BCG (a live attenuated strain of *Mycobacterium bovis*), which was injected into the shoulder a few days after birth. The enlarged lymph nodes were removed surgically. Biopsy confirmed the presence of acid-fast bacilli, consistent with mycobacterial infection. Neither of the patients had presented any other episode of mycobacterial infection by the time of clinical evaluation at three years of age.

### Ethics Statement

This study was conducted in accordance with the Helsinki Declaration, with written informed consent obtained from the parents of P1 and P2 and the other healthy individuals involved. Approval for this study was obtained from the French IRB (*Comité de protection des personnes* or CPP), INSERM and the Rockefeller IRB.

### Epstein-Barr Virus (EBV) Transformation of B Lymphocytes (EBV-B Cells) and Cell Culture

PBMC were isolated from 10 ml of peripheral blood on a Ficoll gradient and were suspended in 4 ml of RPMI1640 supplemented with 20% fetal calf serum (FCS) and 0.2 µg/ml cyclosporine A. We then added 1 ml of EBV medium from the B95.8 cell line [28]. We replaced half the medium every seven days, for three to four weeks, until we observed the formation of clumps of cells. EBV-B cells from the patients were maintained, at a density of 10^6^/ml, in RPMI 1640+10% FCS, 2 mM L-glutamine, 50 units/ml penicillin and 50 µg/ml streptomycin at 37°C. Patient fibroblasts were generated from a skin biopsy sample. Primary fibroblasts were then immortalized by transfection with the SV40 T antigen [29]. They were maintained at subconfluence in Dulbecco's modified Eagle medium (Sigma) supplemented with 10% fetal calf serum, 2 mM L-glutamine, 50 units/ml penicillin and 50 µg/ml streptomycin at 37°C, with passaging (1∶2) every three to four days.

### Exome Sequencing and Analysis

DNA (3 µg) extracted from EBV-B cells from the patient (P1) for massively parallel sequencing was sheared with a Covaris S2 Ultrasonicator (Covaris). An adapter-ligated library was prepared with the Paired-End Sample Prep Kit V1 (Illumina). Exome capture was performed with the SureSelect Human All Exon Kit (Agilent Technologies). Single-end sequencing was performed on an Illumina Genome Analyzer IIx (Illumina), generating 72-base reads. The sequences were aligned with the human genome reference sequence (hg18 build), with BWA aligner [30]. Three open-source packages were used for downstream processing and variant calling: Genome analysis toolkit (GATK), SAMtools and Picard Tools (http://picard.sourceforge.net/). Substitution calls were made with GATK UnifiedGenotyper, whereas indel calls were made with GATK IndelGenotyperV2. All calls with a read coverage ≤4x and a phred-scaled SNP quality of ≤30 were filtered out. All the variants were annotated with the SeattleSeq SNP annotation (http://gvs.gs.washington.edu/SeattleSeqAnnotation/).

### Molecular Analysis

We used National Center for Biotechnology Information (NCBI) accession numbers, including NG_031875.1, NM_001252127.1 and NP_001239056.1 for the number of *AP4E1* genomic DNA (gDNA), mRNA and protein sequences, respectively. Genomic DNA was isolated from the leukocytes of the patients and relatives, as previously described [31]. The 5′-cacagcaactgcaccatttt-3′ and 5′-tggggatctttcttgaccag–3′ primers were used to amplify and sequence *AP4E1* exon 21 and its flanking intron regions. Total RNA from EBV-B cells was used to generate cDNA with the SuperScript III First-Strand Synthesis System (Invitrogen), according to the manufacturer’s protocol. We amplified exons 20 to 24 from the *AP4E1* cDNA with the following primers: Forward primer: 5′-agctg ctggcatcatcatta-3′ and reverse primer: 5′ tggggatctttcttgaccag-3′. Polymerase chain reaction (PCR) was performed with *Taq* polymerase (Applied Biosystems) under the following conditions: 95°C for 10 minutes, 35 cycles of denaturation at 95°C for 30 seconds, annealing at 62°C for 30 seconds and extension at 72°C for 1 min. PCR products were analyzed by electrophoresis in agarose gels, purified on Sephadex, sequenced with the Big Dye Terminator cycle sequencing kit (Applied Biosystems, Foster City, CA) and analyzed on an ABI Prism 3730 (Applied Biosystems, Foster City, CA). We assessed the mRNA levels for the components of the AP-4 complex by reverse transcription- quantitative real-time PCR (RT-qPCR) on an ABI7500. All samples were analyzed in with triplicate, with TaqMan gene expression assays (*AP4B1*, Hs01017576_m1; *AP4E1*, Hs00989109_m1; *AP4M1*, Hs00428091_m1; *AP4S1*, Hs00393029_m1, Applied Biosystems), with *GUS* (β-glucuronidase) gene expression used for the normalization of results.

### Immunofluorescence

Fibroblasts were plated on glass-bottomed dishes (Mattek), fixed with 3% formaldehyde and permeabilized with 0.1% Triton X-100. Cells were labeled with antibodies against AP-4ε (mouse monoclonal BD 612019; raised against amino acids 685–794) and tepsin (rabbit polyclonal [32]), and incubated with Alexa Fluor 594-conjugated anti-mouse and Alex Fluor 488 conjugated anti-rabbit secondary antibodies. All antibodies were used at a concentration of 1 µg/ml. The cells were imaged with a Zeiss Axiovert 200 inverted microscope with a Zeiss Plan Achromat ×63 oil immersion objective, a Hamamatsu ORCA-ER2 camera and IMPROVISION OPENLAB software.

### Western Blotting and Immunoprecipitation

For western blotting, cell pellets were washed in phosphate-buffered saline (PBS), lysed in sample buffer, sonicated and equal amounts of protein were then loaded into the wells of a gel, for electrophoresis. For immunoprecipitation under native conditions, cell pellets containing equal amounts of protein were washed in PBS and then lysed in 1% IGEPAL in PBS supplemented with protease inhibitors. Insoluble material was removed by centrifugation at 20,000×*g* for 20 min, and the sample was precleared with protein-A Sepharose. Immunoprecipitation was then performed with antibodies against subunits of AP-4 and the immune complexes were then isolated with protein-A sepharose. SDS–PAGE and western blots were performed by standard methods with antibodies against various AP-4 subunits [11]. All antibodies were used at a concentration of 1 µg/ml. The polyclonal antibody against AP-4ε was raised in-house against amino acids 607–717. The bands were detected by enhanced chemiluminescence (ECL) and quantified with IMAGEJ software (http://rsb.info.nih.gov/ij).

### Whole-blood Assay of the IL-12/IFN-γ Circuit, Electrophoretic Mobility Shift Assay (EMSA) and Oxidative Burst Test

Whole-blood assays were performed as previously described [33]. Briefly, whole blood diluted 1∶2 in RPMI was either left unstimulated or was stimulated with BCG or BCG+IFN-γ. Supernatants were recovered 48 h later and the levels of IL-12p40 and IL-12p70 production were assessed by ELISA. EMSA was carried out as previously described [21], [23]. Briefly, EBV-B cells were either left unstimulated or were stimulated with various doses of IFN-γ or IFN-α for 20 min. Nuclear proteins were extracted and quantified by the Bradford method. We then incubated 10 µg (stimulated with IFN- γ) or 5 µg (stimulated with IFN-α) of nuclear proteins with a radiolabeled GAS probe corresponding to the Fc-γR1 promoter and subjected the mixture to electrophoresis in a polyacrylamide gel. Oxidative burst assays were performed on EBV B cells as previously described [34].

## Results

### Identification of AP4E1 Deficiency in P1 and P2

We investigated twins (P1 and P2) born to first-cousin Moroccan parents with HSP associated with mycobacterial disease (Figure 1A). We performed WES on genomic DNA from P1 to identify the underlying genetic etiology. We found only one homozygous nonsense mutation in *AP4E1* (Figure 1B, Table 2), resulting in the replacement of an arginine residue in position 1105 with a premature stop codon (p.R1105X). The homozygous mutation was confirmed by Sanger sequencing on gDNA isolated from EBV-B cells and leukocytes (Figure 1C). We collected and sequenced gDNA from peripheral blood samples from other family members and found that both P1 and P2 carried the same homozygous mutation, whereas the parents were heterozygous and a healthy sibling was wild-type at this locus (Figure 1A). This variant was not found in the sequences of 1,050 healthy controls from 51 ethnic groups from the *Centre d'Étude du Polymorphisme Humain* (CEPH) panel [35], [36] or in an additional 69 Moroccan controls. Furthermore, no polymorphic premature stop codon in *AP4E1* was reported in the databases of the NCBI and the 1000-genome project. These results suggest that the *AP4E1* variant is not a polymorphism, but is instead a rare mutation segregating with autosomal recessive disease in this kindred.

**Table 2:** Summary of whole-exome sequencing results.

Type	No. of variants	hom	Het	Total Novel^a^	Novel (hom^b^)	Novel (het^c^)
**All variants**	61514	28599	32915	1199	159	1040
**Nonsynonymous**	8569	3386	5183	222	29	193
**Synonymous**	9342	3835	5507	112	9	103
**Stop gained**	78	21	57	7	1	6
**Stop lost**	20	8	12	0	0	0
**Start gained**	190	98	92	0	0	0
**Start lost**	20	11	9	1	0	1
**Splicing mutation**	115	57	58	9	2	7
**Codon insertion/deletion**	121	81	40	6	2	4
**Frameshift**	147	79	68	6	0	6
**UTR-5** d	167	85	82	2	1	1
**UTR-3** e	525	246	279	13	4	9
**lincRNA** f	129	71	58	3	0	3
**miRNA** g	117	42	75	10	0	10

a Number of variants not found in dbSNP or 1000 Genomes or HapMap and <0.001% in our database;

b Hom: homozygous mutation;

c Het, heterozygous mutation;

d UTR-5: the five-prime untranslated region;

e UTR-3: the three-prime untranslated region;

f lincRNA: long non-coding RNA;

g miRNA: microRNA.

### Molecular Characterization of the *AP4E1* Mutant

**Figure 2:**
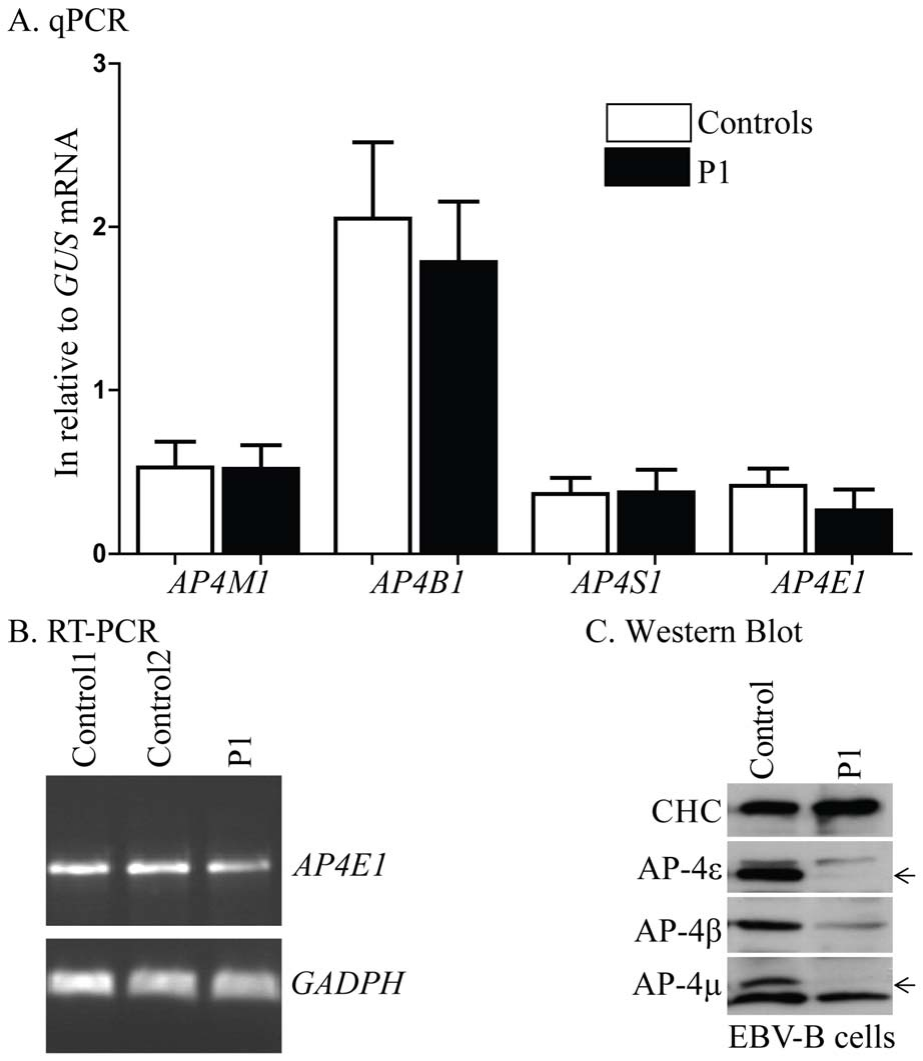
mRNA and protein levels for the subunits of the AP-4 complex. A). RT-qPCR to assess mRNA levels for the components of the AP-4 complex in EBV-B cells from P1. B). RT-PCR to assess the splicing of *AP4E1* mRNA. C). Western blot: whole-cell homogenates from EBV-B cells from P1 and a healthy control were subjected to western blotting for clathrin heavy chain (CHC; loading control), AP-4ε, AP-4β or AP-4 μ. The loss of AP-4ε results in a concomitant decrease in the levels of AP-4β and AP-4 μ (specific bands are indicated by an arrow). These experiments were carried out at least twice.

We then investigated mRNA levels for *AP4E1* and for the other components of the AP-4 complex (*AP4B1*, *AP4M1* and *AP4S1*), by RT-qPCR, in EBV-B cells from P1. The levels of mRNA for *AP4E1*, *AP4B1*, *AP4M1* and *AP4S1* did not differ significantly between cells from P1 and control cells (expressed relative to GUS; Figure 2A). The portion of the cDNA corresponding to exons 20 to 24 of *AP4E1* was also amplified and no aberrant splicing form of *AP4E1* mRNA was found in the cells of P1 (Figure 2B). Together, the RT-qPCR and RT-PCR results suggest that the homozygous p.R1105X mutation had no qualitative or quantitative effect on *AP4E1* gene transcription, at least in EBV-B cells. We then investigated protein levels by western blotting. AP-4ε was barely detectable in EBV-B cells from P1, but was clearly present in control cells (Figure 2C). In addition, the loss of AP-4ε resulted in a concomitant decrease in the levels of both the AP-4 μ and AP-4β proteins in the patient’s cells. These results suggest that the p.R1105X mutation greatly impairs production of the AP-4ε protein, through effects on either protein stability or translation, and that the AP-4ε subunit plays an important role in maintaining the stability of the other subunits of the AP-4 adaptor complex.

### The AP-4 Adaptor Complex in AP4E1 Deficiency

**Figure 3:**
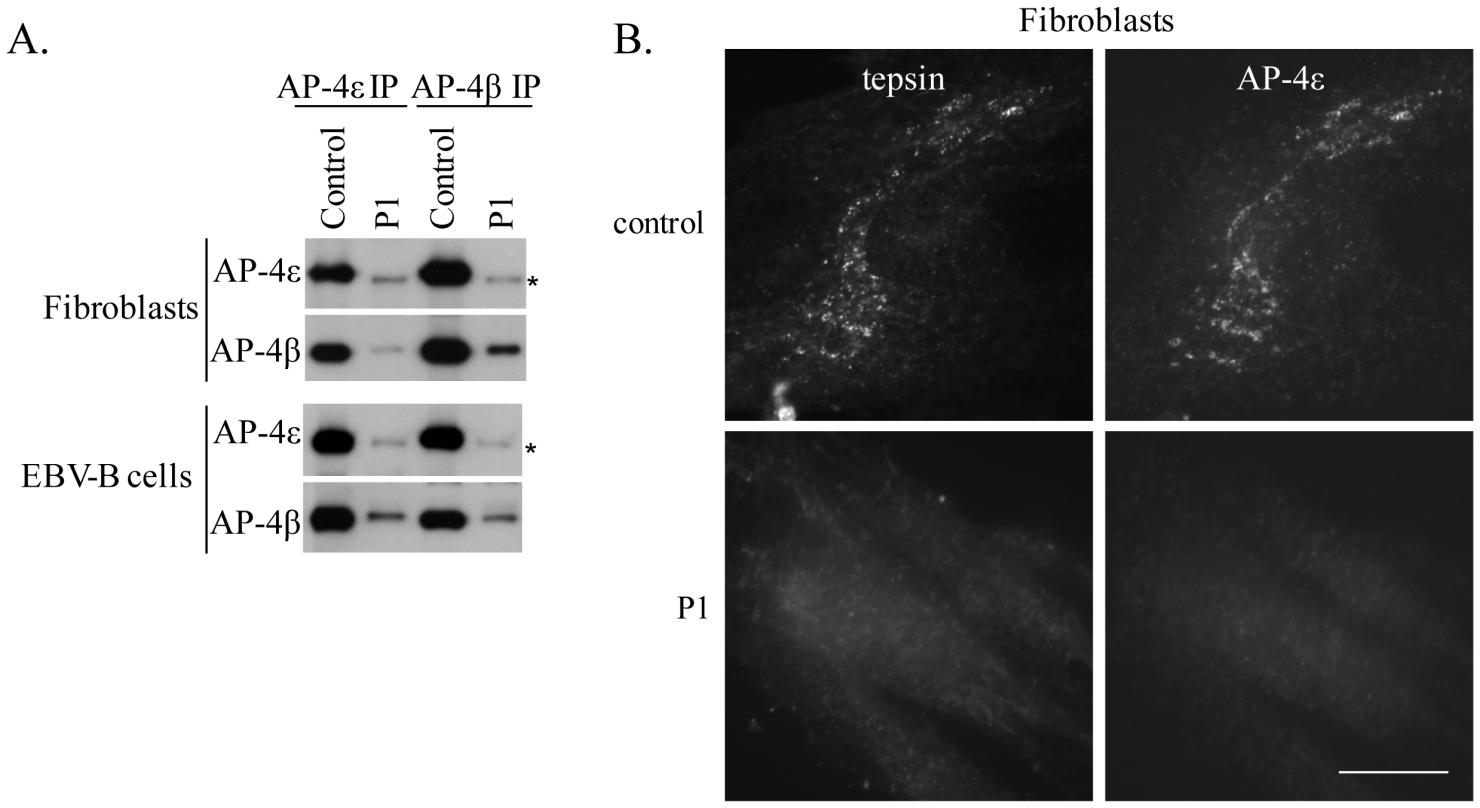
Immunoprecipitation and immunofluorescence of the AP-4 complex in P1. A). EBV-B cells and SV40 fibroblasts from P1 and a healthy control were subjected to immunoprecipitation under native conditions with antibodies against AP-4ε or AP-4β, and the immunoprecipitates were then western blotted with antibodies against AP-4ε or AP-4β. Equal amounts of protein were used for immunoprecipitation from patient and control cells. A small amount of AP-4β is coassembled with AP-4ε, and the AP-4ε assembled with AP-4β in P1 appears to be slightly smaller (AP-4ε*) than the AP-4ε in control cells. B). Fibroblasts from P1 and a healthy control were double-labeled for AP-4ε and the AP-4-associated protein tepsin. Note the specific loss of AP-4ε and tepsin from the cells of P1. Bar 20 µm. These experiments were carried out at least three times.

We also investigated the integrity of the AP-4 complex in EBV-B cells and SV40-transformed fibroblasts from P1, by carrying out native immunoprecipitation with antibodies against AP-4ε or AP-4β. Western-blot analysis showed that the levels of assembled AP-4 were much lower in both fibroblasts and EBV-B cells from P1 than in those from healthy controls (IMAGE J quantification indicated that AP-4 assembly levels were more than 95% lower than those of healthy controls). The residual AP-4ε seemed to be slightly smaller than the corresponding control, possibly reflecting its lower molecular weight, consistent with C-terminal truncation. The loss of AP-4 was confirmed by immunofluorescence staining to detect the AP-4 complex in fibroblasts from P1. In addition, a recently identified AP-4 binding partner, tepsin, which binds to the C-terminal appendage domain of AP-4β [32], was detectable in control fibroblasts, but not in those of P1 (Figure 3B). Overall, we have demonstrated a severe impairment of AP-4 complex formation in both the EBV-B cells and fibroblasts of P1. These results suggest that both patients display autosomal recessive *AP4E1* deficiency, due to an almost complete loss of expression of the AP-4 complex.

### Genetic and Functional Exploration of the IL-12/IFN-γ Pathways

**Figure 4:**
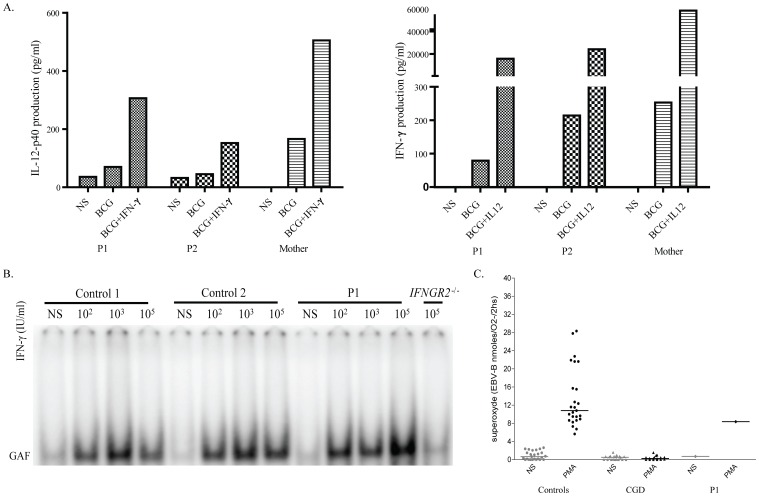
Functional study of the IFN-γ/IL-12 axis and oxidative burst in P1. A). Whole-blood IL-12/IFN-γ pathway screening. P1 and P2 had normal responses to IFN-γ and IL-12. B). EMSA detecting GAF DNA-binding activity after IFN-γ stimulation in the EBV-B cells from two healthy controls, P1 and individuals with complete recessive IFN-γR2 deficiency, used as a negative control. Cells were stimulated for 15 minutes with the indicated dose of IFN-γ. C). Oxidative burst. The production of superoxide in the EBV-B cells from healthy controls, P1 and patients with chronic granulomatous disease, used as negative controls, was determined following stimulation with PMA. These experiments were carried out at least twice.

We first investigated whether the twins (P1 and P2) had any potential immunological abnormalities that might be caused by AP-4 deficiency and would also explain the presence of mycobacterial disease. P1 and P2 had normal counts of neutrophils, monocytes, CD19^+^ B cells, CD3^+^, CD4^+^, CD8^+^ T cells and NK cells. No immunoglobulin or complement defect was found (data not shown). We searched the WES data for mutations in the known MSMD-causing genes (*IFNGR1*, *IFNGR2*, *STAT1*, *IRF8*, *CYBB*, *NEMO*, *IL12RB1*, *IL12B*, *ISG15*). We identified only one heterozygous missense variant in *IFNGR1,* the Q190R mutation. We then carried out functional assays to investigate the IL-12/IFN-γ circuit, which has been shown to be crucial for antimycobacterial immunity [16], [33]. However, the peripheral blood cells of both patients produced normal amounts of IFN-γ in response to stimulation with IL-12 plus BCG, and of IL-12p40 in response to stimulation with IFN-γ plus BCG (Figure 4A). We also searched for any subtle defects in the IFN-γ pathway of the patients [23], [24], [25]. EMSA showed that the levels of IFN-γ-induced gamma-activated factor (GAF) DNA-binding activity in the EBV-B cells from P1 were normal, ruling out the possibility of the *IFNGR1* variant being deleterious (Figure 4B). Furthermore, superoxide production was normal in EBV-B cells from P1, ruling out mutations in genes involved in the phagocyte NADPH oxidase pathway (Figure 4C) [34]. Autosomal dominant *IL12RB2* deficiency has also recently been shown to be responsible for severe primary TB [37]. A heterozygous rare variant was identified in exon 16 of *IL12RB2* in P1. This missense mutation replaced a lysine residue with an asparagine residue at position 768 (K768N). However, this variant was found to have no functional impact in EBV-B cells transduced with the mutant allele and in fresh blood cells stimulated with IL-12 [37]. We were therefore unable to identify any abnormal immunological phenotype able to explain directly the mycobacterial disease in the patients.

### Search for Other Potential Disease-causing Mutations by WES

As mycobacterial disease has not been reported in other patients with AP-4 deficiency, it remained possible that another genetic disorder in the twins would explain the immunological phenotype. We continued to explore this possibility by carrying out WES analysis for P1. We detected 29 homozygous, novel, nonsynonymous mutations in P1, one homozygous nonsense mutation (in *AP4E1*), two homozygous mutations affecting splicing and two homozygous insertions/deletions (indels) (Table 2). However, none of these 34 mutations were considered likely to be related to the clinical phenotypes observed. Only four of these variants affected proteins involved in the immune system: peptidoglycan recognition protein 3 (*PGLYRP3*; R225Q/R225Q), SLAM family member 6 (*SLAMF6*; K71Q/K71Q), granzyme M (*GZMM*; S207L/S207L), leukocyte immunoglobulin-like receptor subfamily B (with TM and ITIM domains) member 1 (*LILRB1*; P496L/P496L). Polyphen and HumVar analysis predicted the *PGLYRP3*, *SLAM6* and *LILRB1* mutations to be benign, and the GZMM S207L/S207L mutation to be probably damaging [38], [39], [40]. However, no link between these molecules and mycobacterial infections has ever been reported. Thus, only the *AP4E1* mutation appeared to be a good candidate for the causal mutation responsible for mycobacterial disease.

## Discussion

The neurological phenotypes in our study, together with those in five other independent studies [3]–[7], are highly consistent, suggesting that these patients can be considered to have “AP-4 deficiency syndrome” [4], a subtype of HSP. In total, 27 patients from nine kindreds, including nine with *AP4E1* mutations, nine with *AP4B1* mutations, six with *AP4S1* mutations, and three with *AP4M1* mutations [4], [5], [6], [7], [8], have a uniform clinical phenotype of type I complex HSP, characterized by severe intellectual disability, microcephaly, progressive spastic paraplegia, growth retardation and a stereotypical laugh. WES-based diagnosis should therefore be considered to check for suspected mutations affecting the AP-4 complex in patients with similar clinical phenotypes. Furthermore, WES on a single identical twin is both a reasonable and practical approach to genetic diagnosis. HSP is characterized by a length-dependent distal axonopathy of the corticospinal tracts [1], [2]. Axons crossed by corticospinal and lower motor neurons may extend for up to 1 m in length and their axoplasm comprises >99% of the total cell volume. Complex intracellular machineries are required for the sorting and distribution of proteins, lipids, mRNA, organelles and other molecules over such long distances [1], [2], [9]. The biological basis of AP-4 deficiency remains unclear, but the severity of the phenotype suggests that AP-4 plays a unique role in a very specific pathway acting on a specific cargo or its sorting. Indeed, it has been reported that AP-4 plays a key role in polarized protein trafficking in neurons [41], and has a neuroprotective function in Alzheimer’s disease [42]. AP-4 has been shown to interact with the transmembrane AMPA glutamate receptor regulatory proteins (TARPs) [41], the δ2 orphan glutamate receptor [43], and amyloid precursor protein [42], although the basis of these interactions and their physiological relevance in HSP are not understood.

The most difficult question with which we are faced here is whether AP-4 deficiency can cause the immunological abnormalities accounting for mycobacterial disease in these patients. First, the co-occurrence of neurological disorders and primary immune deficiency is not uncommon [44]. Indeed, patients with a mutation affecting the related AP-3 complex (*AP3B1* mutation, OMIM*603401), or Hermansky-Pudlak syndrome 2 (HPS2) [45], [46], have recently been reported to present with both recurrent bacterial infections and developmental delay, poor balance and intention tremor [47]. Second, the clinical penetrance of mutations, in terms of infectious diseases can be low, as observed in MSMD for patients with complete IL-12Rβ1 deficiency [20], or even extremely low, as in patients with autosomal dominant IFN-γR2 deficiency [25]. In addition, at least four *AP4E1*-deficient patients are known not to have been vaccinated with live BCG and the vaccination histories of the other three patients are unknown (personal communications from Dr. Colleaux and Dr. Martin). Nonetheless, one patient with an *AP4M1* mutation [8] died from pneumonia at a very young age, although the causal pathogen was not clearly identified. Third, we identified no genetic or functional defects in the known MSMD-causing genes or IFN-γ/IL-12 axis related genes, and the other homozygous mutations do not appear to be realistic candidates. Fourth, it is also possible that clinical phenotype depends on the nature of the mutation concerned and the predicted functionality of the complex. Most of the mutations in *AP4E1* are probably virtually null mutations, due to early truncations or a charge substitution in a critical folded domain [4], [5], [6], [7], [8]. In our case, the nonsense mutation p.R1105X, which is located close to the normal translation termination codon (p.X1137), does not generate a transcript subject to nonsense-mediated mRNA decay (NMD) [48]. This escape from NMD may lead to the production of a C-terminally truncated protein, which may display a dominant-negative activity or a deleterious gain of function. No functional assays for AP-4 are currently available for further investigations of the potential differences between patients with AP-4 deficiencies. We thus propose that the *AP4E1* mutation is the most likely cause of the mycobacterial disease in our patients. The identification of more AP-4-deficient patients and detailed characterization of their clinical and immunological features are required for a full understanding of the role of AP-4 in neurons and, possibly, in immune cells.
